# Métastase mammaire d'un carcinome médullaire de la thyroïde

**DOI:** 10.11604/pamj.2015.22.85.7796

**Published:** 2015-10-01

**Authors:** Asma Gargoura, Naziha Khammassi

**Affiliations:** 1 Faculté de Médecine de Tunis, Service de Médecine Interne, Hôpital Razi, la Manouba 2010, Tunisie

**Keywords:** Cancer médullaire de la thyroïde, métastase mammaire, diagnostic, Medullary thyroid cancer, breast metastasis, diagnostic

## Image en medicine

Le cancer médullaire de la thyroïde (CMT) est un cancer rare des cellules C de la thyroïde. Il représente entre 5 et 10% des cancers thyroïdiens. La diffusion lymphophile du CMT est de survenue fréquente et précoce et la progression métastatique est souvent de localisations multiples, avec atteinte hépatique préférentielle suivie par les poumons et l'os. Le CMT métastase exceptionnellement au sein. Nous rapportons un cas inhabituel de métastase mammaire secondaire d'un CMT se manifestant après 3 ans d’évolution de la tumeur primitive. Patiente âgée de 27 ans, ayant eu une chirurgie radicale pour un carcinome médullaire de la thyroïde. Dans le cadre d'un bilan de surveillance, un scanner thoracique fait neuf mois après la chirurgie avait objectivé une formation médiastinale de 3 cm, qui a été traitée par radiothérapie médiastinale. L’évolution a été marquée par une augmentation progressive du taux de calcitonine avec apparition d'une adénopathie sus claviculaire gauche et de multiples masses mammaires au niveau des deux seins. La mammographie avait montré un amas de microcalcifications rétroaréolaires dont la biopsie mammaire confirmait leur origine métastatique. Les autres diagnostics évoqués devant l'aspect mammographique étaient l'hyperplasie canalaire ou lobulaire, le carcinome intra-canalaire et les lésions bénignes. Le reste du bilan d'extension avait objectivé de multiples métastases osseuses, pulmonaires, hépatiques et pancréatiques. La patiente avait reçu une chimiothérapie à base d'adriamycine et de sels de platine associée à des biphosphonates. Devant l'absence de réponse tumorale, elle a été mise sous traitement palliatif et des soins de support.

**Figure 1:**
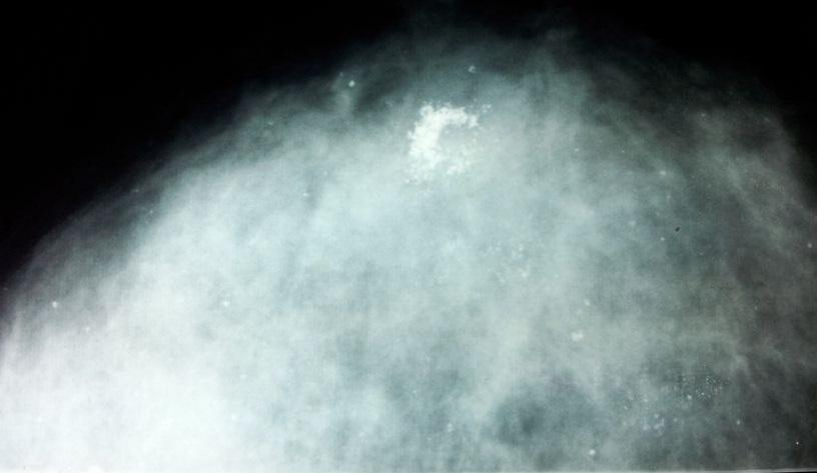
Mammographie: amas de microcalcifications rétroaréolaires

